# Regioselective Oxidation of D-Galacturonic Acid to Provide Crystallized Mucic Acid Using Engineered *Gluconobacter oxydans*

**DOI:** 10.3390/biotech15020040

**Published:** 2026-05-30

**Authors:** Emmeran Bieringer, Lisa Pütthoff, Arne Zimmermann, Ekaterina Burkhanova, David Mijačević, Armin Ehrenreich, Wolfgang Liebl, Dirk Weuster-Botz

**Affiliations:** 1 Chair of Biochemical Engineering, School of Engineering and Design, Technical University of Munich, Boltzmannstraße 15, 85748 Garching, Germany; 2 Chair of Microbiology, School of Life Sciences, Technical University of Munich, Emil-Raman-Straße 4, 85354 Freising, Germany

**Keywords:** whole-cell, biocatalysis, *Gluconobacter oxydans*, multideletion strain, bio-oxidation, galactaric acid, mucic acid, galacturonic acid

## Abstract

Mucic acid (MA) is used as a chelating agent or as a building block for bio-based polymers. MA can be produced by regioselective oxidation of D-galacturonic acid (GA). *Gluconobacter oxydans* is known for the partial oxidation of various substrates via membrane-bound dehydrogenases. As the wild-type strain shows only low oxidation activity towards GA, the engineered multideletion strain *G. oxydans* BP9.1 pta-mGDH, overexpressing a membrane-bound glucose dehydrogenase from *Pseudomonas taetrolens*, was used in buffered whole-cell batch biotransformations with GA as the sole substrate. Initial cell-specific MA formation rates elevated with rising educt concentrations up to 63 g L^−1^. At pH 4, full GA conversion was only achieved with an initial GA concentration of 10 g L^−1^. Complete conversion of 94 g L^−1^ of GA was achieved at pH 5 with 3.4 g L^−1^ of *G. oxydans* BP9.1 pta-mGDH within 48 h, resulting in >100 g L^−1^ of MA, corresponding to a yield of >99% (mol/mol). Isolation of MA (purity > 90%) was achieved after cell separation, followed by cooling crystallization and drying, with a yield of 94%. Complete, full-yield GA conversion using non-growing cells of engineered *G. oxydans* in simple phosphate buffer yielded high product concentrations and enabled simple, high-yield product isolation, thus resulting in cost-effective and sustainable bioproduction of MA.

## 1. Introduction

Mucic acid (MA), or galactaric acid, belongs to the group of aldaric acids. Aldaric acids are polyhydroxy dicarboxylic acids with two terminal carboxylic groups [1]. These two functional groups and the resulting physicochemical properties open a wide range of applications for MA as a platform chemical for use in the cosmetics, food, pharmaceutical, and chemical industries.

Having two carboxyl groups, MA finds usage as a chelating agent [2,3]. Moreover, MA has been extensively studied as a building block for various carbohydrate-based polymers. For instance, MA serves as a precursor of 2,5-furandicarboxylic acid (FDCA) [4,5,6,7,8,9], which is a monomer of bio-based polyesters. Another promising example is the chemical conversion of MA into adipic acid [10,11,12,13] or adipate esters [14], used as repeating units in the manufacturing of biological nylon. Additionally, the utilization of MA for the synthesis of pyrrole-2,5-dicarboxylic acids (PDCAs) [15], carbohydrate-based polyesters [16], MA-containing polyanhydrides [13], or polyamides [17] has been described.

Among other things, its role as a monomer in various polymer structures allows numerous potential applications in the pharmaceutical industry. Notably, three-dimensional lactic acid scaffolds incorporating MA have demonstrated enhanced bone healing capabilities [18]. Moreover, the utilization of MA polymers has facilitated the development of a potential drug delivery system, which has already been tested in vivo in mice to assess its systemic circulation [19]. Additionally, MA can be converted into 2-pyrones, which can serve as polymer building blocks and potentially find applications in the pharmaceutical industry [20]. Lastly, an MA lactone linked to gallate exhibits anti-inflammatory and anti-proliferative effects [21].

Recently, enhancement of the photophysical properties of semiconductors through surface treatment with MA was described [22].

MA can be obtained through the oxidation of galactose, lactose, or galactans using nitric acid or via biological oxidation of galacturonic acid (GA) using isolated enzymes or whole cells [2,23]. Because GA, a pectin monomer, is highly abundant in plant waste residues, many approaches for the production of MA have already been studied (Table 1).

Mojzita et al. engineered *Aspergillus niger* and *Trichoderma reesei* strains to produce MA from GA by deleting D-galacturonate reductases and expressing bacterial uronate dehydrogenases, achieving extracellular MA concentrations of up to 5.9 g L^−1^ over 160 h in a stirred-tank bioreactor (STR) [27]. By applying a fed-batch strategy with lactose as a co-substrate, up to 20 g L^−1^ MA could be produced within ~8 d with an engineered *T. reesei* strain [27,29]. Scale-up of this fed-batch process to the 250 L scale was investigated, resulting in a final MA concentration of 15 g L^−1^ after 7 d [28]. Another approach disrupting the endogenous pathway of GA and the expression of uronate dehydrogenases in marine *Trichoderma* sp. resulted in 25 g L^−1^ MA in fed-batch processes within 7 d [25], which could be enhanced up to 53 g L^−1^ [26].

The same genetic engineering strategy worked for *Escherichia coli*. Disruption of uronate isomerase and D-galactarate dehydrogenase, simultaneously expressing a uronate dehydrogenase, enhanced the production of MA from GA to 10.3 g L^−1^ over a period of 48 h in a shake-flask culture [10].

Other promising approaches for producing MA use *Saccharomyces cerevisiae*. Benz et al. expressed a GA transporter from *Neurospora crassa* and an uronate dehydrogenase in *S. cerevisiae* to facilitate intracellular MA formation [35]. An engineered *S. cerevisiae* strain co-utilizing glucose and GA was able to produce 8 g L^−1^ MA from orange peel waste within 80 h in shake flasks, introducing a GA transporter from *A. niger* [3]. By applying protein engineering to an uronate dehydrogenase from *S. cerevisiae*, product concentration could be increased 13-fold to 3.35 g L^−1^ after 48 h in shake flasks compared to the wild-type enzyme [33]. Recently, bioconversion of citrus peel waste into 20 g L^−1^ MA within 120 h was observed in stirred-tank bioreactors using an uronate dehydrogenase-overexpressing *S. cerevisiae* strain and xylose as co-substrate [31]. Prototrophic *S. cerevisiae* were engineered for nitrogen-independent growth and enabled the conversion of citrus waste into 15.4 g L^−1^ MA during 48 h of shake-flask cultivation [32].

One of the latest studies used engineered *Pseudomonas putida* expressing a membrane-bound glucose dehydrogenase (mGDH) to yield up to 22.4 g L^−1^ of MA after 24 h in shake flasks [34].

Most current bio-oxidation approaches for MA synthesis suffer from low final product concentrations, the need for co-substrates to balance redox, and the need for efficient substrate and product transporters in the cell membrane. The use of *Gluconobacter oxydans* as a highly efficient whole-cell biocatalyst could overcome these limitations through its unique properties which make it attractive for industrial applications, e.g., the manufacturing of vitamin C using the Reichstein–Grüssner process [36,37] or the conversion of glycerol to the self-tanning agent dihydroxyacetone [38,39]. *G. oxydans* is able to perform incomplete oxidations across a broad substrate spectrum, e.g., sugars and acids, via membrane-bound dehydrogenases [40,41]. Thus, no transport of substrates and products through the cell membrane is necessary. In addition, this Gram-negative acetic acid bacterium is known for its high tolerance to osmotic pressure, which enables the use of high substrate concentrations and, therefore, could lead to high product concentrations.

Resting cells of wild-type *G. oxydans* have already been used for the oxidation of GA to produce MA, achieving concentrations as low as 2.6 g L^−1^ within 72 h. This biotransformation works best at pH 4. However, conversion was low (~50%) when using pure GA [24]. Although most MA bioproduction methods (Table 1) used an uronate dehydrogenase for the conversion of GA into MA, the GA oxidation activity of glucose dehydrogenases has already been observed [34,42,43].

Therefore, we decided to use the multideletion strain *G. oxydans* BP9.1, which has nine native mDHs deleted [44], and to overexpress a membrane-bound glucose dehydrogenase from *Pseudomonas taetrolens* for the regioselective oxidation of GA into MA. This engineered *G. oxydans* strain has already shown high cellobiose oxidation activity, resulting in >500 g L^−1^ cellobionic acid at >99% conversion using resting cells in scalable batch processes in stirred-tank bioreactors [45].

Bioprocess design should consider the whole bioprocess: upstream (biotransformation) and downstream processing (product isolation). The costs for product isolation can be minimized if resting cells are used in an aqueous buffer without any other organic additives, e.g., yeast extract, and full conversion of the substrate without byproduct formation can be achieved at high final product concentrations. Therefore, we aimed to identify the key process variables for the efficient batch production of MA in whole-cell biocatalysis with non-growing cells of recombinant *G. oxydans* in scalable stirred-tank bioreactors to achieve full GA conversion and high final MA concentrations and yield: initial GA concentration, pH, and initial biocatalyst concentration.

A few MA isolation methods have already been described [10,27,31], making use of the low solubility of MA in water at low pH [23]. For example, cells were separated via centrifugation, followed by an acidification of the supernatant with HCl, which induced the crystallization of MA. Crystallization could be enhanced by freezing and thawing the solution. The crystals were recovered by centrifugation and then (vacuum-) dried. Overall, both a purity and yield of >97% could be achieved [27]. Based on this, we aimed to isolate MA after whole-cell biotransformation and cell separation through acidification of the product solution followed by cooling crystallization and drying of the MA crystals.

## 2. Materials and Methods

### 2.1. Materials

D-galacturonic acid monohydrate and galactaric acid, with a purity of ≥97% each, were ordered from Sigma-Aldrich Chemie GmbH (Taufkirchen, Germany). All other chemicals were purchased from Carl Roth GmbH & Co. KG (Karlsruhe, Germany).

### 2.2. Bacterial Strains

The multideletion strain *Gluconobacter oxydans* BP9.1 pta-mGDH derives from the wild-type *G. oxydans* 621H (DSM 2343). A membrane-bound glucose dehydrogenase from *Pseudomonas taetrolens* was expressed from an expression plasmid in the multideletion chassis strain *G. oxydans* BP9, constructed by Peters et al. [44,46]. The construction of the expression plasmid is based on the work of Mientus et al. [47]. Details on *G. oxydans* BP9.1 pta-mGDH have been published recently [45].

The wild-type strain *G. oxydans* 621H was purchased from the German Collection of Microorganisms and Cell Cultures (DSMZ) (Braunschweig, Germany).

### 2.3. Maintenance

*G. oxydans* cells were kept in 35% (*v*/*v*) glycerol at −80 °C. A single colony from an agar plate was used to inoculate 50 mL of sterile pre-culture medium in shake flasks without baffles (see Section 2.4). Each strain was cultivated for at least 48 h at 30 °C and 180 rpm (Multitron, Infors HT, Bottmingen, Switzerland). After mixing of the incubated cells with glycerol, the suspension was aliquoted into sterile 1.5 mL reaction tubes. After incubation for 1 h at room temperature, glycerol stocks were preserved at −80 °C for subsequent use.

### 2.4. Preparation of Pre-Cultures

The pre-culture of the wild-type *G. oxydans* 621H and *G. oxydans* BP9.1 pta-mGDH was carried out in two stages [45,48]. For both stages, a medium containing 5 g L^−1^ yeast extract, 3 g L^−1^ peptone from casein, and 10 g L^−1^ fructose (FYP medium) was used [49]. The pH was adjusted with 3 M HCl to 6.0. FYP medium was autoclaved for 20 min at 121 °C before use. After sterilization, the sterile-filtered (0.22 µm, Minisart^®^ High Flow, Sartorius AG, Göttingen, Germany) antibiotics cefoxitin and kanamycin were added to reach a final concentration of 50 mg L^−1^. For the incubation of the wild-type culture, only cefoxitin was used to prevent contamination.

To start the first stage, 50 mL FYP medium in sterile 500 mL shake flasks without baffles was inoculated with 500 µL of the glycerol cell stock. The cells were incubated for at least 72 h at 30 °C and 180 rpm in a rotary shaking incubator. The second stage was performed in sterile 1 L shake flasks with baffles. A volume of 200 mL FYP medium was inoculated with 10% (*v*/*v*) of the cell suspension from the first stage of the pre-culture. After incubation at 30 °C for 24 h at 150 rpm, cells were harvested for further use.

The cell suspension of four 1 L shake flasks was separated by centrifugation in a sterile 1 L centrifugation flask (3260× *g*, 20 min, 30 °C, Rotixa 50 RS, Andreas Hettich GmbH & Co. KG, Tuttlingen, Germany). The pellets were resuspended in sterile phosphate-buffered saline solution (PBS) containing 8.0 g L^−1^ NaCl, 0.2 g L^−1^ KCl, 1.42 g L^−1^ Na_2_HPO_4_, and 0.27 g L^−1^ KH_2_PO_4_ (pH 7.4) and combined into a single 50 mL centrifugation tube. After a second centrifugation step (3260× *g*, 20 min, 30 °C), the pellet was resuspended in 50 mL of the medium for biomass production (see Section 2.5) and loaded into a sterile syringe (BD Discardit II, Becton Dickinson, Franklin Lakes, NJ, USA) equipped with a sterile single-use cannula (Sterican 0.8 × 120 mm, B. Braun, Melsungen, Germany) for inoculation of the stirred-tank bioreactor for biocatalyst production.

### 2.5. Production of G. oxydans Whole-Cell Biocatalysts

The production of *G. oxydans* 621H and *G. oxydans* BP9 pta-mGDH for use as whole-cell biocatalysts was studied in controlled 7.5 L STRs operated in batch mode (Labfors, Infors-HT, Bottmingen, Switzerland) as described in detail previously [45]. The bioreactor was provided with sensors for online monitoring of pH (EasyFerm Plus PHI Arc 425, Hamilton AG, Bonaduz, Switzerland) and dissolved oxygen (DO) (VisiFerm DO ECS 425, Hamilton AG, Bonaduz, Switzerland). Furthermore, the temperature and concentrations of oxygen and carbon dioxide in the off-gas were measured online. A constant temperature was maintained at 30 °C through tempered water cycling in a double jacket. During cultivation, automatic titration with 25% (*v*/*v*) NH_4_OH enabled pH 6.0 to be maintained by using the peristaltic pump of the bioreactor setup. Sufficient oxygen supply to the cells was ensured by maintaining the DO concentration above 30% of air saturation, achieved by elevating the agitation rate from 500 to 800 rpm. Sterile-filtered air (0.2 µm, Acro^®^ 50, Pall Filtersystems GmbH, Crailsheim, Germany) was dispersed at a constant flow rate of 1 vvm by three six-blade Rushton turbines assembled at the axis of the bioreactor. The off-gas was sterile-filtered before passing through the exhaust-gas analyzer (BlueVary, BlueSens Gas Sensor GmbH, Herten, Germany). To prevent evaporative effects, the STR was configured with an off-gas cooler. The control script for temperature, DO and pH was provided by the proprietary control software of the bioreactor (Eve^®^ 2021 H1R1, Infors-HT, Bottmingen, Switzerland).

A modified complex medium according to Buchert et al. was used for biocatalyst production containing 5 g L^−1^ yeast extract, 3 g L^−1^ peptone from casein, 5 g L^−1^ (NH_4_)_2_SO_4_, 0.5 g L^−1^ MgSO_4_· × 7 H_2_O, 1 g L^−1^ KH_2_PO_4_, 1.5 g L^−1^ K_2_HPO_4_, and 0.1% (*v*/*v*) antifoam (Antifoam 204, Sigma-Aldrich Chemie GmbH, Taufkirchen, Germany) [50]. 50 g L^−1^ D-glucose was used for the cultivation of the multideletion strain *G. oxydans* BP9.1 pta-mGDH, while 50 g L^−1^ D-mannitol was used for the wild-type strain. Prior to steam sterilization, the pH probe was calibrated at pH 4 and pH 7. The STR, charged with the complex medium containing neither D-glucose nor D-mannitol, MgSO_4_ × 7 H_2_O, and antifoam, was steam-sterilized at 121 °C for 20 min. Stock solutions of 400 g L^−1^ D-glucose, 100 g L^−1^ D-mannitol, 250 g L^−1^ MgSO_4_ × 7 H_2_O, and 10% (*v*/*v*) antifoam (Antifoam 204, Sigma-Aldrich Chemie GmbH, Taufkirchen, Germany) were sterilized separately and added after autoclaving according to the concentrations above. Sterile-filtered cefoxitin was added to the medium via a septum using sterile single-use syringes with sterile cannulas to obtain a target concentration of 2.5 mg L^−1^ in the fermentation medium. Additionally, 50 mg L^−1^ of sterile-filtered kanamycin was applied in the production of *G. oxydans* BP9.1 pta-mGDH. The DO probe and the exhaust-gas analyzer were calibrated at 100% of air saturation and 20.97% O_2_ and 0.04% CO_2_, respectively, after the medium was saturated by gassing with 1 vvm of sterile air and stirring at 500 rpm for at least 20 min. The pH 6.0 was manually adjusted with 12.5% (*v*/*v*) H_3_PO_4_.

The bioprocess started with the inoculation with *G. oxydans* (initial OD_600_~0.2) from the pre-culture (see Section 2.4). Cell growth was monitored offline (see Section 2.8). After reaching the stationary phase within 24 h (wild type: 48 h), cells were harvested by centrifugation. Therefore, the cell suspension was pumped from the reactor into a sterile 5 L flask and then aliquoted into four sterile 1 L centrifugation flasks. After centrifugation (3260× *g*, 30 min, 30 °C), the supernatants were discarded, and the pellets were washed with ~200 mL sterile PBS (pH 7.4) and unified in one 1 L centrifugation flask for the second centrifugation step (3260× *g*, 15 min, 30 °C). The supernatant was discarded and the cells were resuspended in biotransformation medium without the substrate. The biocatalyst suspension was loaded into sterile 20 mL syringes equipped with sterile single-use cannulas to achieve the desired initial catalyst concentration in the subsequent biotransformations.

### 2.6. Biotransformations in Stirred-Tank Bioreactors

Bio-oxidation experiments were performed in a fully controlled parallel lab-scale STR system (DASGIP^®^ Bioblock, DASGIP^®^ reactor SR0700ODSS, Eppendorf AG, Hamburg, Germany). Temperature, pH, DO, and the off-gas concentration of O_2_ and CO_2_ were monitored for each STR. The temperature was kept constant at 30 °C and pH was maintained at the desired value (pH 4–pH 6) by titrating 25% (*v*/*v*) NH_4_OH or 12.5% (*v*/*v*) H_3_PO_4_ using the micro-peristaltic pumps of the parallel bioreactor setup. Oxygen supply was ensured by dispersion of sterile air with two six-blade Rushton turbines assembled at the axis of each bioreactor. DO concentration was maintained at ≥30% of air saturation by adapting the stirrer speed between 500 and 1200 rpm. Each bioreactor was equipped with an exhaust-gas cooler to prevent evaporation during bioprocesses. Off-gas was sterile-filtered before passing through the exhaust-gas analyzer (EasyLine, ABB, Zurich, Switzerland or BlueVary, BlueSens Gas Sensor GmbH, Herten, Germany). The process control software of the bioreactor system was used for control of pH, temperature, and DO (Dasgip Control 4.5, Eppendorf AG, Hamburg, Germany).

Biotransformation experiments were conducted with resting cells of *G. oxydans* in a phosphate buffer modified by Burger et al. containing 1.0 g L^−1^ KH_2_PO_4_, 1.6 g L^−1^ Na_2_HPO_4_, 1.4 g L^−1^ (NH_4_)_2_SO_4_, 0.2 g L^−1^ MgSO_4_· × 7 H_2_O, 0.1 g L^−1^ CaCl_2_ × 2 H_2_O, and 0.1% (*v*/*v*) antifoam 204 (Sigma-Aldrich Chemie GmbH, Taufkirchen, Germany) [48]. Different GA and biocatalyst concentrations were set for each experiment during preparation. The initial reaction volume ranged from 0.3 to 0.5 L.

The channels of the micro-peristaltic pumps for each STR were disinfected by rinsing with 70% (*v*/*v*) ethanol, 2 M NaOH, and sterile deionized water. Then, the lines were filled with 25% (*v*/*v*) NH_4_OH, and 12.5% (*v*/*v*) H_3_PO_4_, respectively. Before assembling the pH sensors (405-DPAS-SC-K8S, Mettler-Toledo International Inc., Columbus, OH, USA), a 2-point calibration at pH 4 and pH 7 was performed. The bioreactors were filled with ~500 mL deionized water and steam-sterilized at 121 °C for 20 min. Stock solutions of D-galacturonic acid ranging from 60 to 130 g L^−1^ were sterile-filtered into a sterile flask using a bottle-top filter (0.22 µm, Steritop^®^ PES Membran, Merck KGaA, Darmstadt, Germany) and a vacuum pump (LABOPORT^®^, KNF Neuberger GmbH, Freiburg, Germany) to prevent thermal denaturation during autoclaving. After autoclaving, the deionized water was discarded and the components of the biotransformation medium were filled into the reactor under a sterile bench (HERA SAFE KS 12, Thermo Electon LED GmbH, Langenselbold, Germany). A ten-fold stock solution of KH_2_PO_4_, Na_2_HPO_4_, and (NH_4_)_2_SO_4_ was sterilized separately. Stock solutions of 250 g L^−1^ MgSO_4_· × 7 H_2_O, 250 g L^−1^ CaCl_2_ × 2 H_2_O, antifoam, D-galacturonic acid solution and the stock of KH_2_PO_4_, Na_2_HPO_4_ and (NH_4_)_2_SO_4_ were combined according to the concentrations mentioned above. When using the multideletion strain *G. oxydans* BP9.1 pta-mGDH as biocatalyst, sterile-filtered kanamycin was added via a septum, using sterile single-use syringes with sterile cannulas, reaching a concentration of 50 mg L^−1^. A 2-point calibration was performed for the DO sensors (VisiFerm DO ECS 225, Hamilton AG, Bonaduz, Switzerland) and the off-gas analyzer. Therefore, the bioreactors were stripped with sterile air (DO = 100% of air saturation, O_2_ concentration = 20.97%, CO_2_ concentration = 0.04%) as well as with sterile nitrogen (DO = 0%, O_2_ concentration = 0%, CO_2_ concentration = 0%). The pH was manually adjusted to the desired value with 12.5% (*v*/*v*) H_3_PO_4_ or 25% (*v*/*v*) NH_4_OH. To initiate biotransformations, resting *G. oxydans* prepared as described in Section 2.5 were added to the medium.

### 2.7. Isolation of MA

Cooling crystallization followed by drying was applied for the isolation of MA after cell separation. When the batch bio-oxidation of GA to MA was finished, 25% (*v*/*v*) NH_4_OH was added to achieve pH 8.5 using the micro-peristaltic pumps of the bioreactor setup [31]. After the MA was fully dissolved after 10 min, the fermentation broth was harvested. The dissolution of the MA crystals was checked microscopically (Axioplan, Carl-Zeiss Jena GmbH, Jena, Germany). MA was harvested through centrifugation in non-sterile 1 L centrifugation flasks (3260× *g*, 30 min, 30 °C) to separate cells from the product solution. Then, the supernatant was filtered through a bottle-top filter. Next, 3 M HCl was added to achieve pH 1.3, and the solution was frozen at −20 °C for 24 h to promote MA crystallization [10,27]. After thawing the mixture for at least 30 min at RT, the supernatant was removed through centrifugation (3260× *g*, 30 min, 30 °C) and the remaining crystallized MA was dried in a drying oven for at least 24 h at 50 °C (KB 115, Binder GmbH, Tuttlingen, Germany). Purity was measured by HPLC. The yield was determined gravimetrically.

### 2.8. Offline Analytics

To track cell dry weight (CDW) as well as substrate and product concentrations, samples were taken from the bioreactors during biotransformation and/or biomass production experiments. These samples were then analyzed offline.

Measurements of the optical density at 600 nm (OD_600_) in 1 cm single-use cuvettes using a single-beam photometer (Genesys 10S UV–VIS, Thermo Scientific, Neuss, Germany) were used to estimate CDW concentrations. A technical triplicate was measured for each sample, which had to be diluted in PBS to be in the linear range of the photometer (0.1–0.3). Linear correlation factors were used according to Burger et al. to assess the CDW from OD_600_ (biomass production: 0.36 g L^−1^; biotransformation: 0.48 g L^−1^) [48].

The GA and MA concentrations were quantified using high-performance liquid chromatography (HPLC) analysis (LC-2030C Plus, Shimadzu Corp., Kyoto, Japan) as previously described [27,28,31,51]. To analyze the concentrations of dissolved GA and MA, samples were prepared as follows: Samples from biotransformation experiments with cell suspensions were centrifuged at room temperature (14,500× *g*, 10 min, Espresso, Thermo Fisher Scientific Inc., Waltham, MA, USA) in 1.5 mL reaction tubes to separate the liquid phase from the cells. Additionally, the supernatant was filtered (0.22 µm, Chromafil RC20/15 MS, Macherey-Nagel GmbH & Co. KG, Düren, Germany) into clean 1.5 mL reaction tubes to remove residual cells and solid particles. The samples were either measured immediately or stored at 4 °C until HPLC analysis.

If MA had already crystallized in the reactor, a second version of each HPLC sample was prepared to quantify the total concentration of the formed product. The cell suspension, including the MA crystals, was diluted 1:20 in 0.1 M NaOH to achieve pH 9 in 1.5 mL reaction tubes, vortexed until complete dissolution of MA, and centrifuged (14,500× *g*, 10 min, RT). The supernatant was filtered through a size-exclusion filter (0.22 µm, Chromafil RC20/15 MS, Macherey-Nagel GmbH & Co. KG, Düren, Germany) to remove remaining cells and was analyzed immediately by HPLC.

The HPLC was equipped with an ultraviolet (UV) detector (PD-20A/20AV, Shimadzu Corp., Kyoto, Japan) operating at 210 nm. An anion exchange column was used as the stationary phase (Aminex HPX-87H, Bio-Rad Laboratories Inc., Hercules, CA, USA). The column temperature was set to 60 °C. Isocratic elution was carried out at a constant flow rate of 0.6 mL min^−1^ using 2.5 mM H_2_SO_4_ as the mobile phase. The injection volume was 10 µL and chromatograms were recorded within 22 min. The anion exchange column was operated at 48 bar. Standard measurements of GA and MA were performed separately over the range of 0.5–8 g L^−1^ to provide references for samples with unknown concentrations. Reference samples for MA were prepared in 0.1 M NaOH to ensure all particles dissolved, while GA was diluted in deionized water. All unknown samples were diluted with either water or 0.1 M NaOH to fall within this range. The retention times of MA and GA were ~7.7 min, and ~8.4 min, respectively.

### 2.9. Determination of the Oxygen Uptake Rates

The concentrations of CO_2_ and O_2_ in the off-gas of each bioreactor were measured to calculate the oxygen uptake rates (OUR). Equation (1) was used, comprising a reaction volume V_R_, a molar volume of 22.414 L mol^−1^ (V_mol_), an air flow rate F_Air_, an inlet and outlet fraction of O_2_ and CO_2_ ($y_{{\mathrm{CO}}_{2}}$ and $y_{O_{2}}$) and the inert gas factor θ (Equation (2)). Therefore, equal gas flow rates (in-gas and off-gas) were assumend.

(1) $$\mathrm{OUR}=\frac{F_{\mathrm{Air}}}{V_{R}\cdot V_{\mathrm{mol}}}\cdot \left(y_{O_{2}}^{\mathrm{in}}-\theta \cdot y_{O_{2}}^{\mathrm{out}}\right)$$

(2) $$\theta =\frac{1-y_{{CO}_{2}}^{in}-y_{O_{2}}^{in}}{1-y_{{CO}_{2}}^{out}-y_{O_{2}}^{in}}$$

### 2.10. Determination of Volumetric and Biomass-Specific MA Formation Rates

The determination of the volumetric (Q_MA_) and biomass-specific MA formation rates (q_MA_) was conducted by analyzing the progress curves of the product concentrations obtained from the batch biotransformation experiments. Therefore, MA concentration curves were fitted to Equation (3) with the MA concentration c_MA_, the model parameter k, and the process time t using the SciPy package (Version 1.10.1) in a Python environment (Version 3.10). Parameter fitting of c_MA,max_ and k was realized with the function scipy.optimize.curve_fit, which was based on the method of minimizing the sum of squared errors. From the replicated experiments, product concentrations were fitted globally. For the non-replicated experiments single progress curves were fitted. The 95% confidence band of non-linear as well as linear regressions was calculated from the covariance matrix of the estimated parameters by applying uncertainties from the model predictions.

The volumetric MA formation rate as a function of process time was calculated using Equation (4) and the fitted model parameters. To estimate the initial biomass-specific MA formation rate (q_MA,max_), the volumetric MA formation rate was divided by the initial biocatalyst concentration (c_X0_) according to Equation (5).

(3) $$c_{\mathrm{MA}}\left(t\right)\;=c_{\mathrm{MA},\max}\cdot \left(1-e^{-k\cdot t}\right)$$

(4) $$Q_{\mathrm{MA}}\left(t\right)=\frac{{\mathrm{dc}}_{\mathrm{MA}}}{\mathrm{dt}}=c_{\mathrm{MA},\max}\cdot k\cdot e^{-k\cdot t}$$

(5) $$q_{\mathrm{MA},\max}=\frac{Q_{\mathrm{MA},0}}{c_{X0}}$$

## 3. Results and Discussion

### 3.1. Production of MA with Resting G. oxydans Cells

**Figure 1:**
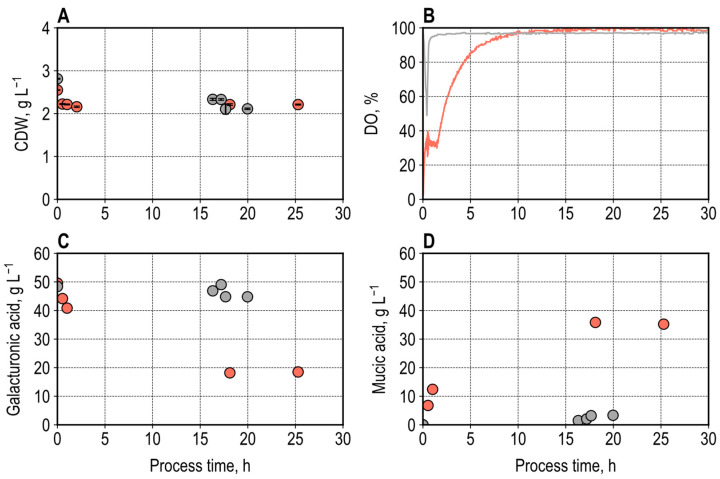
Regioselective bio-oxidation of 50 g L^−1^ GA with non-growing cells of recombinant *G. oxydans* BP9.1 pta-mGDH (grey) compared to the wild-type *G. oxydans* 621H (orange) for the formation of MA in a STR: (**A**) CDW concentration, (**B**) DO concentration, (**C**) galacturonic acid concentration, and (**D**) mucic acid concentration as function of process time. Error bars in (**A**) represent the standard deviation of the OD measurement in technical triplicates (V_R_ = 0.4 L, pH 4.0, T = 30 °C, F_air_ = 2.0 vvm, DO > 30% of air saturation by increasing the stirrer speed *n* = 500–770 rpm).

In a previous study, pH 4 was found to be the most suitable for producing mucic acid with resting cells of *G. oxydans* using extracted GA from pectin [24]. Accordingly, pH 4 was chosen for the initial bio-oxidations. Figure 1 shows the results of two different batch biotransformations of GA to MA using *G. oxydans* in fully controlled stirred-tank bioreactors. The oxidation activity towards ~50 g L^−1^ galacturonic acid of the multideletion strain *G. oxydans* BP9.1 pta-mGDH was compared to the wild-type *G. oxydans* 621H.

The initial cell density of the multideletion strain was 2.6 g L^−1^. The biocatalyst concentration decreased rapidly after inoculation by 15% to 2.2 g L^−1^ and stayed constant until the end of the batch process after 24 h (Figure 1A). GA conversion and MA formation began immediately after inoculation (Figure 1C,D). Due to the high oxygen demand of the cells caused by the high oxidation rate, the DO concentration decreased initially (Figure 1B) with a maximum oxygen uptake rate of 47 mM h^−1^. It declined with increasing process time, reaching almost zero once bio-oxidation stopped after ~12 h (Figure S1 in the Supplementary Materials). The initial decrease in biomass concentration may be explained by a short period of about seven minutes, where DO dropped below 20% air saturation at the beginning of the batch process due to a delayed dynamic response of the DO controller (delayed increase in the stirrer speed). The agitation rate was increased to 770 rpm to maintain the DO concentration above 30% of air saturation (Figure 1B). After 1.5 h, the stirring speed was restored to the initial 500 rpm and DO began to increase, reaching 97% of air saturation after 12 h. Finally, a substrate conversion rate of 63% was achieved. No byproducts could be measured (σ_P_ ≥ 99% (mol/mol)). Based on the reported dissociation constants of MA at comparable temperatures (pK_a1_ ≈ 2.96–3.08; pK_a2_ ≈ 3.63–3.97), it was mainly present as mucate at pH 4 [52,53,54,55].

The initial CDW concentration of the wild-type strain was 2.8 g L^−1^ and this was reduced by 25% to 2.1 g L^−1^ during the bio-oxidation (Figure 1A). Compared to the multideletion strain, only a small amount of oxygen was required, resulting in a drop in DO to 50% of air saturation at the start of the biotransformation, followed by an immediate increase to 95% of air saturation (Figure 1B). Only 3.3 g L^−1^ MA was formed by applying an initial GA concentration of 48 g L^−1^ and ending at a concentration of 44.8 g L^−1^ (Figure 1C,D), which resulted in a selectivity σ_P_ of ~84% (mol/mol). However, compared to previously published results on the production of MA with *G. oxydans* NL71, the final concentration was 29% higher, while using only 28% of the biocatalyst concentration [24].

To conclude, applying the multideletion strain in batch bio-oxidations of GA improved the final MA concentration by a factor of 10.6, demonstrating the superior MA production capacity of the multideletion strain of *G. oxydans* overexpressing the mGDH from *P. taetrolens*. This could be attributed to the higher oxidation activity of the mGDH from *P. taetrolens* compared to that of the native mGDH from *G. oxydans*, which could be explained by either a higher specific activity or a higher amount of expressed enzyme due to overexpression. Comparable effects were already described for the production of L-erythrulose with a similar multideletion strain, overexpressing a membrane-bound polyol-DH [48].

### 3.2. Variation of Initial GA Concentration

**Figure 2:**
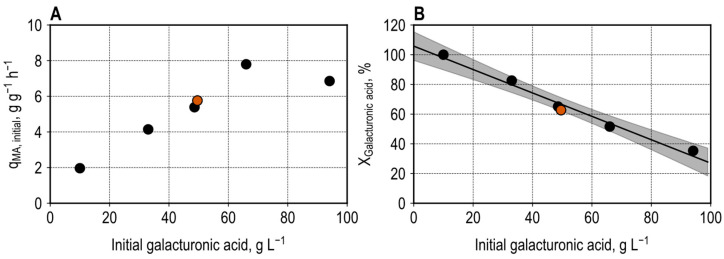
Initial biomass-specific MA formation rates (**A**) and the conversion of GA (**B**) over the initial GA concentrations in regioselective batch bio-oxidations with non-growing cells of *G. oxydans* BP9.1 pta-mGDH in STRs. The grey area represents the confidence interval (+/− of three standard deviations). MA formation and GA conversion with *G. oxydans* BP9.1 pta-mGDH derived from Figure 1 are highlighted in orange (●) (c_X0_ = 2.6 g L^−1^, pH 4.0, T = 30 °C, F_air_ = 2.0 vvm, V_R0_ = 0.3–0.4 L, DO > 30% of air saturation ensured by controlling the stirrer speed *n* between 500 and 915 rpm).

The initial biomass-specific MA formation rates (q_MA_) and the GA conversion are shown as a function of the initial substrate concentration in batch bio-oxidations with non-growing cells in Figure 2. Increasing substrate concentrations of c_GA,0_ = 10 g L^−1^, 33 g L^−1^, 49 g L^−1^, 50 g L^−1^, 65 g L^−1^, and 95 g L^−1^ resulted in higher MA formation rates of 2.0 g g^−1^ h^−1^, 4.1 g g^−1^ h^−1^, 5.4 g g^−1^ h^−1^, 5.8 g g^−1^ h^−1^, 7.8 g g^−1^ h^−1^, and 6.9 g g^−1^ h^−1^, respectively (MA progress curves and volumetric MA formation rates can be found in Figure S2 in the Supplementary Materials). The initial MA formation rates increased linearly with increasing initial substrate concentration up to 65 g L^−1^ GA. However, with 95 g L^−1^ GA, the initial MA formation rate was considerably reduced (Figure 2A), which may indicate the onset of slight substrate inhibition or saturation of the mGDH binding sites.

All batch experiments showed a product selectivity σ_P_ of > 99% (mol/mol) with no byproduct formation over a process time of 24 h. Although the initial reaction rate increased with rising initial substrate concentrations (c_GA,0_), the relative conversion of GA decreased from 100% to 83%, 65%, 63%, 52%, and 35% (Figure 2B). This effect may occur because pH 4 may not be the most favourable pH for *G. oxydans* at higher GA concentrations. The process time during which the biocatalysts were actively converting the substrate was insufficient to achieve higher conversion rates when combining low pH and high initial GA concentrations. Applying these reaction conditions (2.6 g L^−1^ *G. oxydans* BP9.1 pta-mGDH, pH 4.0, batch process time of 24 h), complete oxidation of GA was only achieved at c_GA,0_ = 10 g L^−1^, and the highest initial MA formation rate of 7.8 g g^−1^ h^−1^ was measured at an initial GA concentration of 65 g L^−1^.

### 3.3. Variation of Initial G. oxydans BP9.1 pta-mGDH Concentration

**Figure 3:**
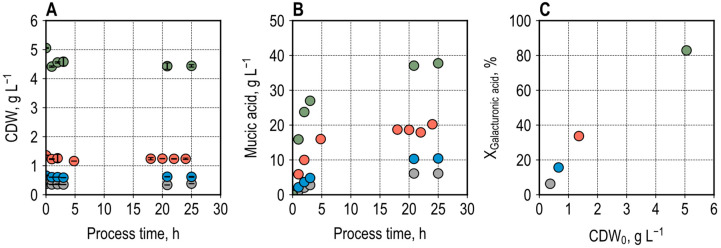
CDW (**A**) and MA (**B**) concentrations as a function of process time, and GA conversion as a function of initial CDW concentrations (**C**) in biotransformations with resting cells of *G. oxydans* BP9.1 pta-mGDH in stirred-tank bioreactors with varying initial CDW concentrations: c_X0_ = 0.4 g L^−1^ (●), c_X0_ = 0.7 g L^−1^ (●), c_X0_ = 1.4 g L^−1^ (●), and c_X0_ = 5.0 g L^−1^ (●). Error bars in (**A**) represent the standard deviation of the OD measurements in technical triplicates (c_S0_ = 45 g L^−1^, pH 4.0, T = 30 °C, F_air_ = 2.0 vvm, V_R_ = 0.4 L, DO > 30% of air saturation ensured by increasing stirrer speed n = 500–1000 rpm).

A higher initial biocatalyst concentration should enable higher GA conversion in batch experiments with fixed process time. Therefore, batch biotransformations with varying initial CDW concentrations of 0.4–5.0 g L^−1^ *G. oxydans* BP9.1 pta-mGDH were conducted, applying an initial GA concentration c_GA,0_ of 45 g L^−1^, each (Figure 3), which is in a comparable range of the initial GA concentration of the reference process in Section 3.1.

With increasing initial biocatalyst concentrations, higher final product concentrations were measured within 24 h (Figure 3B). The conversion of GA increased linearly with increasing initial whole-cell biocatalyst concentration until 1.4 g L^−1^ *G. oxydans* BP9.1 pta-mGDH (Figure 3C). However, at the highest initial biomass concentration of 5.0 g L^−1^, only 83% conversion was achieved instead of the expected 100%. In this batch process, MA crystallization was visible in the bioreactor at the end. In batch processes with initial biomass concentrations of 1.4 g L^−1^ or higher, a slight decrease in *G. oxydans* cells by ~15% was observed (Figure 3A).

DO concentration and OUR were measured to rule out possible oxygen limitations. Even at the highest initial CDW concentration, no oxygen limitation was observed, as an increase of stirrer speed up to 1000 rpm was sufficient to keep DO above 30% of air saturation. After an initial decline in DO concentrations, marking the beginning of substrate oxidation, DO saturated at ≥95% of air saturation in every batch process within 24 h, indicating the termination of bio-oxidation (see Figure S3 in the Supplementary Materials). To summarize, increasing the initial cell density up to 5.0 g L^−1^ led to a nearly linear increase in the relative GA conversion, but even with the highest applied CDW, 100% substrate conversion was not achieved.

### 3.4. Bio-Oxidation of GA at Varying pH

**Figure 4:**
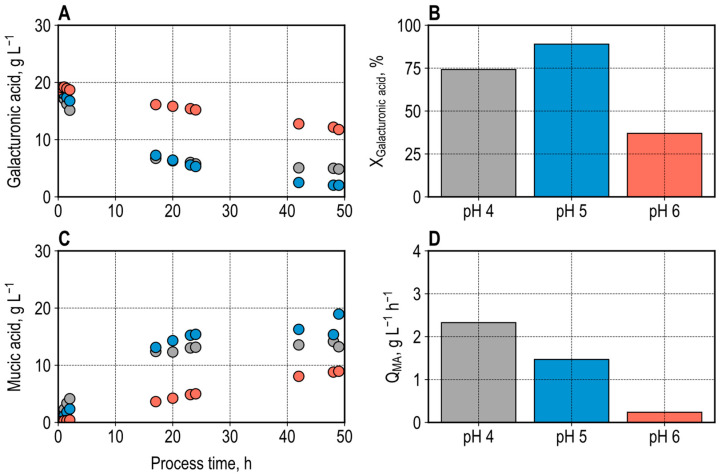
GA (**A**) and MA (**C**) concentrations as a function of process time, GA conversion (**B**), and initial volumetric MA formation rates (**D**) in bio-oxidations with non-growing cells of *G. oxydans* BP9.1 pta-mGDH in STRs at pH 4 (●), pH 5 (●), and pH 6 (●) (c_X0_ = 1.6 g L^−1^, c_S0_ = 19 g L^−1^, T = 30 °C, F_air_ = 2.0 vvm, V_R_ = 0.5 L, DO > 30% air saturation).

So far, complete GA conversion has been achieved only at low initial GA concentrations (10 g L^−1^) in batch processes with *G. oxydans* BP9.1 pta-mGDH at pH 4.0 within 24 h (Figure 2B). Efficient bio-oxidation of cellobiose was achieved with the same whole-cell biocatalyst at pH 6.0 [45]. Therefore, additional batch bio-oxidation processes were performed at varying pH and with a prolonged process time of 48 h, with nearly double the initial GA concentration, at 19 g L^−1^. The objective of the study was to determine whether an increase in pH positively affects GA conversion rate, independent of initial cell density and GA concentration. Figure 4A and Figure 4C show the GA and MA concentrations at pH 4.0, pH 5.0, and pH 6.0, respectively, clearly demonstrating pH 5.0 is the best option, with the highest conversion of 89.1% within 48 h at an initial whole-cell biocatalyst concentration of 1.6 g L^−1^ (Figure 4C). However, the highest initial volumetric formation rate of 2.3 g L^−1^ h^−1^ MA was observed at pH 4 (Figure 4D), which corresponded to the highest initial oxygen consumption rate of 11.9 mM h^−1^ (see Figure S4 in the Supplementary Materials), but only 13.7 g L^−1^ MA was produced over 48 h (74% conversion).

The initial volumetric MA formation rate at pH 5.0 was reduced to 1.5 g L^−1^ h^−1^ compared to the MA formation rate at pH 4.0. This is also reflected by the reduced initial OUR of 7.6 mM h^−1^ at pH 5. However, the bio-oxidation activity of *G. oxydans* BP9.1 pta-mGDH was very much prolonged compared to pH 4.0. No decline in oxygen consumption to zero was observed within 48 h (see Figure S4 in the Supplementary Materials).

Carrying out bio-oxidations of GA at pH 5 instead of pH 4 enabled higher GA conversion rates. As proton gradients, which are generated through mDH electron transfer, play a crucial role in the energy metabolism by generating ATP in *G. oxydans* [41], pH 5 could be more favourable and therefore oxidation of galacturonic acid was prolonged compared to pH 4.

### 3.5. Towards High MA Concentrations at Complete GA Conversion

**Figure 5:**
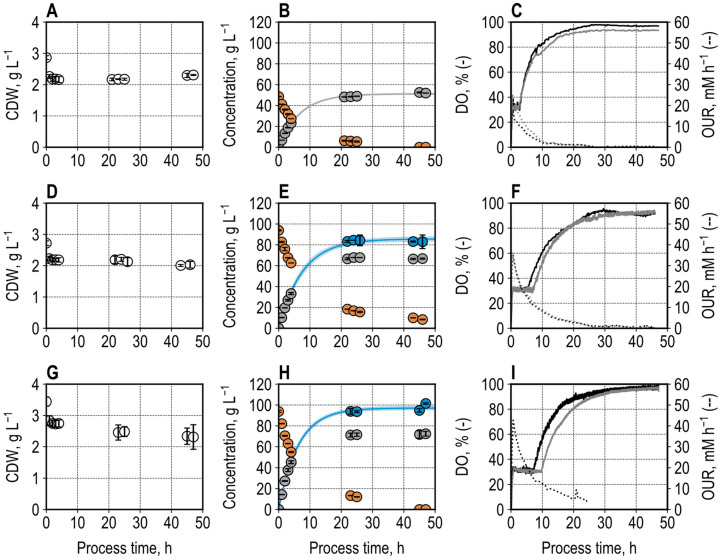
CDW (**A**,**D**,**G**), GA (●), and soluble MA (●) concentrations (**B**,**E**,**H**), and DO concentration (solid line) and OUR (dotted line) (**C**,**F**,**I**) as a function of process time in regioselective bio-oxidations with non-growing *G. oxydans* BP9.1 pta-mGDH in STRs. The batch processes were reproduced once, min–max values are indicated with error bars in (**A**,**B**,**D**,**E**,**G**,**H**), and duplicates are shown in black and grey in (**C,F**,**I**). The grey and blue areas (**B**,**E**,**H**) indicate the confidence interval (+/−) of three standard deviations of the non-linear regression of mucic acid concentrations. In (**E**,**H**), the total product concentration, including the crystalized fraction of MA, is shown in blue (●) (pH = 5.0, T = 30 °C, F_air_ = 2.0 vvm, V_R_ = 0.4 L, DO > 30% of air saturation ensured by increasing stirrer speed, *n* = 500–1030 rpm).

Bio-oxidations with resting cells of *G. oxydans* BP9.1 pta-mGDH were carried out in duplicates at pH 5.0 with step-by-step variation of the initial biocatalyst and GA concentrations to achieve high final MA concentrations and complete GA conversion within a batch process time of 48 h (Figure 5). First, full conversion of ~50 g L^−1^ GA was investigated with ~2.8 g L^−1^ *G. oxydans*. Then, a GA concentration of ~94 g L^−1^ was tested, keeping the initial cell density at ~2.8 g L^−1^. Finally, full conversion was examined with c_GA,0_ = ~94 g L^−1^ and an increase in the concentration of *G. oxydans* up to 3.4 g L^−1^.

An initial biocatalyst concentration of 2.86 ± 0.04 g L^−1^ was sufficient to completely convert 48.6 ± 0.1 g L^−1^ GA within 48 h (Figure 5A–C). The cell density decreased from the start of the bio-oxidation process until it stabilized at approximately 2.2 g L^−1^. MA production was observed immediately after inoculation. This was reflected in the increased OUR. The DO concentration was maintained above 30% of air saturation for 2.4 ± 0.2 h by increasing the stirrer speed to 710 ± 3 rpm (see Figure S5 in the Supplementary Materials). After about 27 h, the DO signal reached saturation at ~95% of air saturation, marking the end of bio-oxidation and complete GA consumption. Accordingly, the OUR decreased from the initial 24.2 ± 8.0 mM h^−1^ to nearly zero. Finally, a MA concentration of 51.9 ± 0.1 g L^−1^ was achieved, corresponding to a yield of 99.4 ± 0.6% (mol/mol) and a substrate conversion of >99.9% (mol/mol). No significant crystallization of MA was observed in the stirred-tank bioreactors.

Subsequently, nearly doubling the initial GA concentration of 93.9 ± 0.5 g L^−1^ was tested at a similar whole-cell biocatalyst concentration of 2.72 ± 0.04 g L^−1^ (Figure 5D–F). CDW concentration decreased immediately after inoculation, falling to 2.20 ± 0.04 g L^−1^ after 24 h, and eventually reaching 2.03 ± 0.07 g L^−1^. With more substrate initially present, the OUR increased. The agitation rate was raised to 816 ± 12 rpm to keep DO above 30% of air saturation. After 5.4 h and 7.1 h, respectively, the stirrer speed returned to the initial 500 rpm. The initial OUR of 37.5 ± 1.1 mM h^−1^ was ~55% higher compared to the lower initial GA concentration. Although no complete GA conversion was observed. After about 24 h, 16.8 ± 0.2 g L^−1^ GA was still present in the stirred-tank bioreactor. At the end of the batch process, 8.4 ± 0.4 g L^−1^ GA remained, corresponding to a substrate conversion of ~91%. The lack of an increase in the solved MA concentrations, despite GA consumption over the last 24 h, may be attributed to the adhesion of crystallized MA to the stirrers. As a result, the product was not detected in the HPLC measurement. After about 20 h, MA crystallization became visible. The final soluble MA concentration was around 68 g L^−1^, while the final total MA concentration was 82.9 ± 6.5 g L^−1^.

Finally, to achieve full conversion at the initial GA concentration of 93.8 ± 1.0 g L^−1^, the initial biocatalyst concentration was increased to 3.44 ± 0.11 g L^−1^ (Figure 5G–I). As before, the cell concentration decreased continuously during the bio-oxidation process, finally reaching 2.31 ± 0.35 g L^−1^. The initially increased cell density resulted in a higher OUR of 47.9 mM h^−1^. Unfortunately, due to a technical defect, only the first 25 h of the OUR could be recorded. Agitation rates of 983 ± 61 rpm were necessary to maintain DO concentrations above 30% of air saturation for the first 7.3 h, and 9.8 h, respectively. After 45 h, no GA was detected in either parallel batch biotransformation. Crystallization of the product MA was observed in the stirred-tank bioreactors after approximately 20 h. As shown before, a soluble concentration of 70 g L^−1^ MA was measured. The final total product concentration was 101.4 ± 0.7 g L^−1^ MA, corresponding to a yield of 99.2 ± 0.7% (mol/mol). In conclusion, increasing the initial whole-cell biocatalyst concentration to ~3.4 g L^−1^ was sufficient for complete GA conversion at pH 5.0, resulting in a final MA concentration of >100 g L^−1^.

In contrast to previous bio-oxidation processes for MA synthesis, the final MA concentration of >100 g L^−1^ we achieved with resting cells of *G. oxydans* BP9.1 pta-mGDH in a simple batch process within 48 h is 191% higher than the so-far published highest MA concentration of 53 g L^−1^ (see Table 1) which was measured in a fed-batch process with marine *Trichoderma* sp. for 7 d expressing a soluble uronate dehydrogenase [26]. The high concentration of MA indicates that biological activity can be maintained over an extended period. Additionally, the efficient conversion may be attributed to two main factors: first, the high stability of the expressed mGDH in the cytoplasmic membrane, and second, the effective electron transfer within the respiratory chain of *G. oxydans*, which is required for GA oxidation and is also used for energy conservation [41]. The significantly reduced reaction time required to achieve complete GA conversion could be explained by the higher specific oxidation activity of the *G. oxydans* cells. Above all, this could be attributed to the absence of substrate and product transport into the cells due to membrane-mediated oxidation. Another advantage of this extra-cytoplasmic oxidation is that no catabolic genes need to be deleted to prevent the metabolism of GA or MA [3,25,29,35]. Furthermore, the application of a fed-batch process may indicate a possible substrate inhibition in *Trichoderma* sp. processes [25,29]. This could be overcome by using *G. oxydans*, which is known for its high osmotolerance.

Cell-specific formation rates, oxygen uptake rates, and product concentrations were lower than those for cellobionic acid production using the multideletion strain BP9.1 expressing the mGDH. Most likely, this is due to reduced substrate affinity for GA compared with the disaccharide cellobiose, which has greater structural similarity to the native substrate because it consists of two β-1,4-linked glucose molecules. Additionally, for the mGDH of *P. taetrolens*, a 18% lower substrate affinity towards galactose, the reduced form of GA, compared to cellobiose, was reported [56].

Since the costs of biomass production are quite high because of the low maximum cell densities of *G. oxydans*, batch growth improvements have recently been published by applying combinations of cheap substrates, like glucose and fructose, in complex medium [57].

### 3.6. Isolation of MA by Crystallization

**Figure 6:**
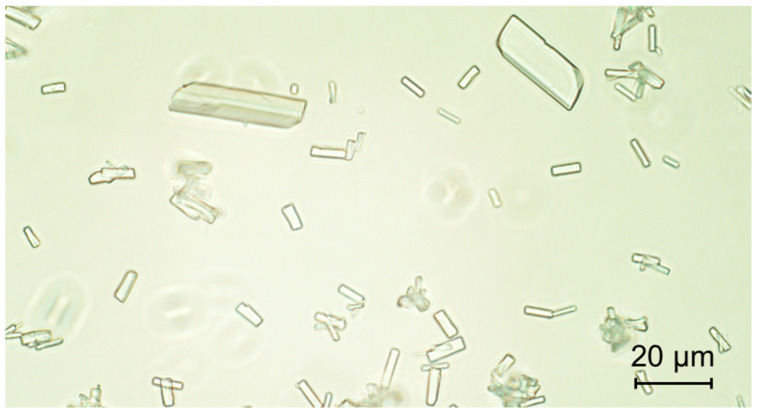
Microscopy image of crystalized MA at pH 1.3 after thawing of the clarified fermentation broth after biotransformation of GA using *G. oxydans* BP9.1. pta-mGDH.

After full GA conversion, NaOH was added to the cell suspension to dissolve the crystallized MA. To ensure full dissolution, samples were examined under a light microscope for residual MA solids. Afterwards, the *G. oxydans* cells were separated by centrifugation. Crystallization of MA was induced by acidifying the supernatant to pH 1.3 and freezing for 24 h at −20 °C. Figure 6 shows single block-shaped MA crystals after thawing of the acidified supernatant. A similar geometry for isolated MA has already been described [26,58]. Because of the acidic environment, MA crystals are present in the pure acid form [52,53,54,55]. Some amorphous aggregates were observed but were not further characterized. After thawing to room temperature, the MA crystals were dried at 50 °C until the weight stabilized. The solids had a purity of 90.1 ± 1.2% MA, and the final yield of the whole downstream processing was 94.0 ± 6.7%. GA was not identified as an impurity in HPLC measurements. Therefore, the impurities are most likely residual salts from the biotransformation buffer.

## 4. Conclusions

Applying resting cells of the multideletion strain *G. oxydans* BP9.1, heterologously expressing a membrane-bound glucose dehydrogenase from *P. taetrolens*, for the bio-oxidation of galacturonic acid enables mucic acid concentrations of up to 100 g L^−1^, achieving yields and selectivities ≥ 99% (mol/mol). Simple batch biotransformations are sufficient for high productivities, as the MA formation rate could be increased by applying high GA concentrations and will remain high over a longer process time. By preventing the formation of byproducts and harvesting after full substrate conversion in phosphate buffers without expensive additives, the costs of both biocatalytic MA production and subsequent purification should be considerably reduced compared to literature.

Additionally, the low nutritional requirements of this multideletion strain in biotransformations with resting cells and the known high tolerance of *G. oxydans* against varying organic compounds are beneficial for the conversion of hydrolyzed GA-rich pectin waste from residual plant materials, e.g., extracted sugar beet press pulp or citrus peel waste [59,60]. Thus, a sustainable biocatalytic process using renewable feedstocks may be realized with *G. oxydans* BP9.1. pta-mGDH to produce value-adding mucic acid within the next few years.

## Figures and Tables

**Table 1:** Comparison of whole-cell biotransformations to produce MA by regioselective oxidation of GA with respect to final product concentrations (c_MA_), scale, and the microorganisms used.

Process	Microorganism	c_MA_, g L^−1^	Scale, L	Reference
Batch ^1^	*Gluconobacter oxydans*	2.52	1	[24]
Batch	*Gluconobacter oxydans*	2.55	1	[24]
Batch ^2^	*Escherichia coli*	10.3	0.05	[10]
Batch ^1,2^	*Escherichia coli*	6.9	0.05	[10]
Fed-batch	*Trichoderma* sp.	25	0.5–2	[25]
Fed-batch	*Trichoderma* sp.	53	0.11	[26]
Batch	*Trichoderma reesei*	5.9	-	[27]
Fed-batch ^1^	*Trichoderma reesei*	18.9	1	[28]
Fed-batch ^1^	*Trichoderma reesei*	21	10	[28]
Fed-batch ^1^	*Trichoderma reesei*	14	250	[28]
Fed-batch ^1^	*Trichoderma reesei*	20	-	[29]
Batch ^1,2^	*Aspergillus niger*	3.1	-	[30]
Batch ^1^	*Saccharomyces cerevisiae*	20	3	[31]
Batch ^1,2^	*Saccharomyces cerevisiae*	8.0	0.05	[3]
Batch ^1,2^	*Saccharomyces cerevisiae*	15.4	0.01	[32]
Batch ^2^	*Saccharomyces cerevisiae*	3.35	0.05	[33]
Batch ^1^	*Pseudomonas putida*	22.4	0.015	[34]
Batch ^1,2^	*Pseudomonas putida*	7.4	0.015	[34]

^1^ (Hydrolyzed) pectin (either commercial or crude) was used as substrate. ^2^ Process was carried out in shake flasks.

## Data Availability

The original contributions presented in the study are included in the article. Further inquiries can be directed to the corresponding author.

## Supplementary Materials

The following supporting information can be downloaded at https://www.mdpi.com/article/10.3390/biotech15020040/s1, Figure S1: Stirrer speed (A) and OUR (B) as a function of process time in bio-oxidations of 50 g L^−1^ GA with non-growing cells of recombinant *G. oxydans* BP9.1 pta-mGDH (red) compared to the wild-type *G. oxydans* 621H (grey) for the production of MA in a STR (V_R_ = 0.4 L, pH 4.0, T = 30 °C, F_air_ = 2.0 vvm, DO > 30% air saturation by controlling the stirrer speed *n* = 500–770 rpm); Figure S2: MA concentration (●), interpolated line for progress fitting (solid line), and volumetric MA formation rates (dashed lines) (A–F) as a function of process time in bio-oxidations with non-growing cells of *G. oxydans* BP9.1 pta-mGDH in STRs applying different initial GA concentrations (c_S0_). Data derived from Figure 1 and Figure S1 are highlighted in orange (●) (c_X0_ = 2.6 g L^−1^, pH 4.0, T = 30 °C, F_air_ = 2.0 vvm, V_R0_ = 0.3–0.4 L, DO > 30% air saturation ensured by increasing stirrer speed *n* = 500–915 rpm); Figure S3: OUR, stirrer speed (grey), and DO concentration (black) in bio-oxidations with non-growing cells of *G. oxydans* BP9.1 pta-mGDH in STRs with different initial CDW concentrations: c_X0_ = 0.4 g L^−1^ (A,B), c_X0_ = 0.7 g L^−1^ (C,D), c_X0_ = 1.4 g L^−1^ (E,F), and c_X0_ = 5.0 g L^−1^ (G,H) (c_S0_ = 45 g L^−1^, pH 4.0, T = 30 °C, F_air_ = 2.0 vvm, V_R_ = 0.4 L, DO > 30% air saturation ensured by increasing stirrer speed, *n* = 500–1000 rpm); Figure S4: CDW concentration, OUR, stirrer speed (grey), and DO concentration (black) as a function of process time in bio-oxidations with non-growing cells of *G. oxydans* BP9.1 pta-mGDH in STRs at pH 4 (A–C), pH 5 (D–F), and pH 6 (G–I). Error bars in (A,D,G) represent the standard deviation of the OD measurements in technical triplicates (c_S0_ = 19 g L^−1^, T = 30 °C, F_air_ = 2.0 vvm, V_R_ = 0.5 L, DO > 30% air saturation); Figure S5: Stirrer speed as a function of process time in bio-oxidations with non-growing *G. oxydans* BP9.1 pta-mGDH in STRs. Different CDWs and initial GA concentrations were applied: CDW_0_ = 2.86 ± 0.04 g L^−1^, c_GA,0_ = 48.6 ± 0.1 g L^−1^ (A); CDW_0_ = 2.72 ± 0.04 g L^−1^, c_GA,0_ = 93.9 ± 0.5 g L^−1^ (B); CDW_0_ = 3.44 ± 0.11 g L^−1^, c_GA,0_ = 93.8 ± 1.0 g L^−1^ (C). The batch processes were reproduced once, duplicates are shown in black and grey (pH = 5.0, T = 30 °C, F_air_ = 2.0 vvm, V_R_ = 0.4 L, DO > 30% air saturation ensured by increasing stirrer speed, *n* = 500–1030 rpm).

## Abbreviations

The following abbreviations are used in this manuscript:

DO	Dissolved oxygen
FYP (medium)	Fructose-yeast extract-peptone (medium)
GA	Galacturonic acid
HPLC	High-performance liquid chromatography
MA	Mucic acid
mGDH	Membrane-bound glucose dehydrogenase
OD_600_	Optical density at 600 nm
OUR	Oxygen uptake rate
PBS	Phosphate-buffered saline solution
STR	Stirred-tank bioreactor
UV	Ultraviolet
vvm	Vessel volume per minute
RT	Room temperature
CDW	Cell dry weight
pta	*Pseudomonas taetrolens*
mDH	Membrane-bound dehydrogenase
