# Supplementary material for: Regioselective Oxidation of D-Galacturonic Acid to Provide Crystallized Mucic Acid Using Engineered Gluconobacter oxydans
Source: BioTech (Basel). 2026 May 30;15(2):40. doi: 10.3390/biotech15020040 (PMC13297029; doi:10.3390/biotech15020040)
Supplement: Supplementary file 1 [file biotech-15-00040-s001.zip › biotech-4303870-supplementary.pdf]

BioTech

Supplementary material

Regioselective Oxidation of D-Galacturonic Acid to Provide Crystallized Mucic Acid Using Engineered *Gluconobacter oxydans*

Emmeran Bieringer [1], Lisa Pütthoff [2], Arne Zimmermann [1], Ekaterina Burkhanova [1], David Mijačević [1], Armin Ehrenreich [2], Wolfgang Liebl [2] and Dirk Weuster-Botz [1,*]

[1] Chair of Biochemical Engineering, School of Engineering and Design, Technical University of Munich, Boltzmannstraße 15, 85748 Garching, Germany
[2] Chair of Microbiology, School of Life Sciences, Technical University of Munich, Emil-Raman-Straße 4, 85354 Freising, Germany
* Correspondence: dirk.weuster-botz@tum.de

Supplementary Materials

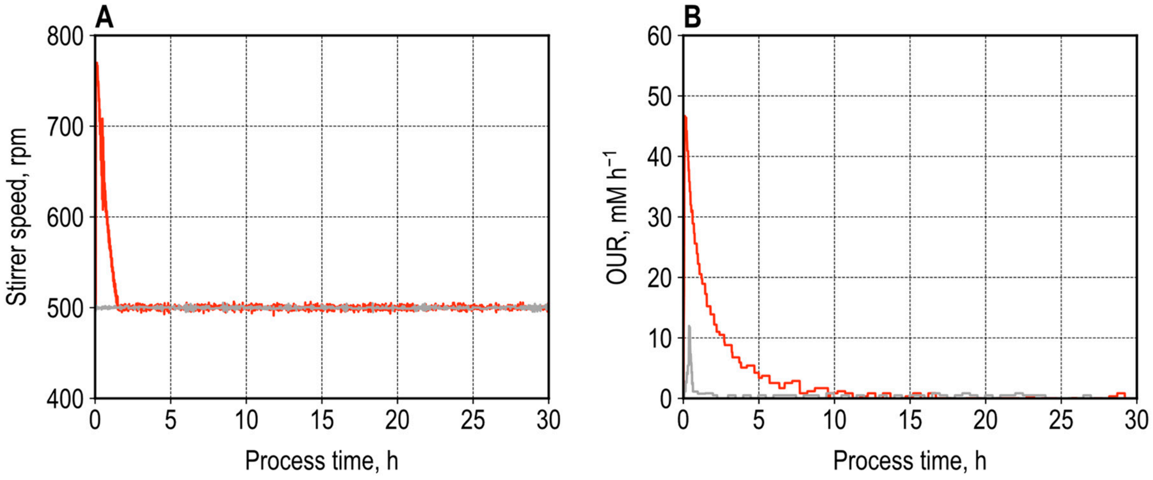

Figure S1. Stirrer speed **(A)** and OUR **(B)** as a function of process time in bio-oxidations of 50 g L^{-1} GA with non-growing cells of recombinant *G. oxydans* BP9.1 pta-mGDH (red) compared to the wild-type *G. oxydans* 621H (grey) for the production of MA in a STR (V_R = 0.4 L, pH 4.0, T = 30 °C, F_{air} = 2.0 vvm, DO > 30% air saturation by controlling the agitation rate *n* = 500–770 rpm).

 https://doi.org/10.3390/biotech15020040

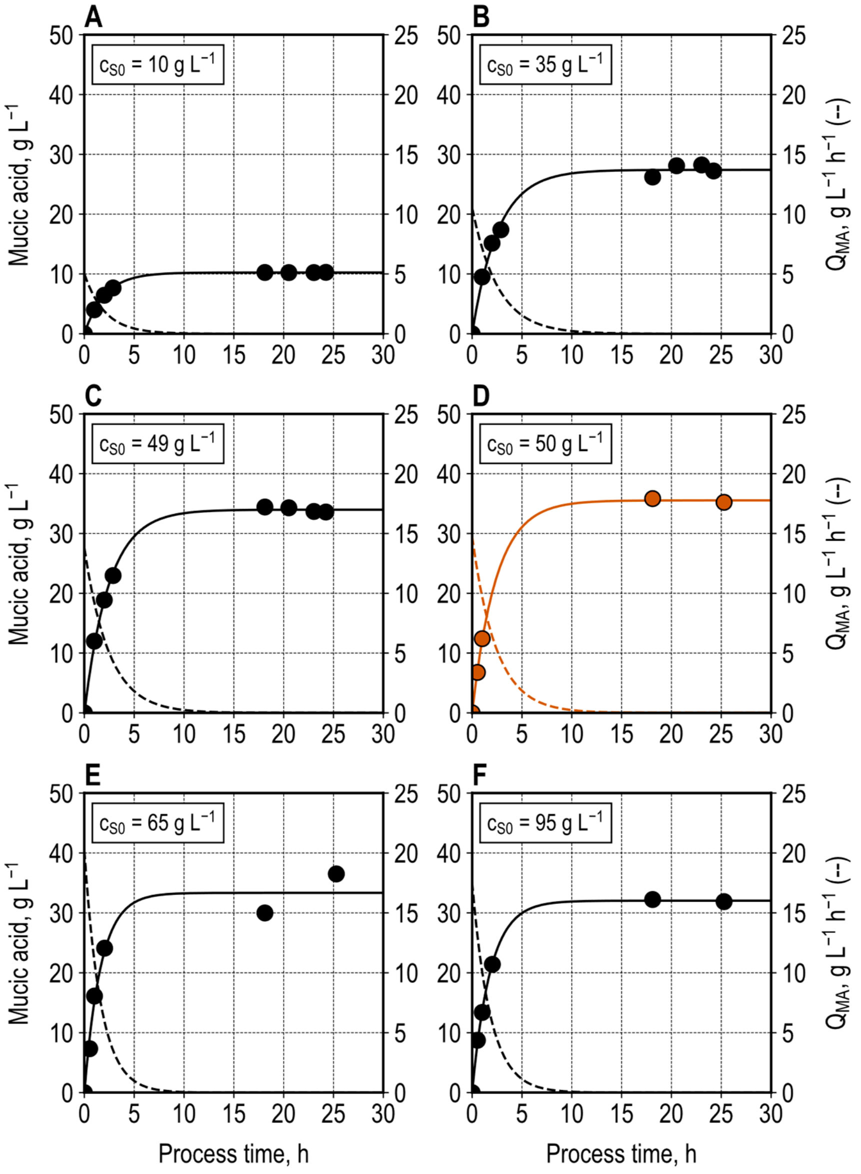

Figure S2. MA concentration (●), interpolated line for progress fitting (solid line), and volumetric MA formation rates (dashed lines) **(A-F)** as a function of process time in bio-oxidations with non-growing cells of *G. oxydans* BP9.1 pta-mGDH in STRs applying different initial GA concentrations (c_{S0}). Data derived from Figure 1 and Figure S1 are highlighted in orange (●) (c_{X0} = 2.6 g L^{-1}, pH 4.0, T = 30 °C, F_{air} = 2.0 vvm, V_{R0} = 0.3–0.4 L, DO > 30% air saturation ensured by increasing stirrer speed n = 500–915 rpm).

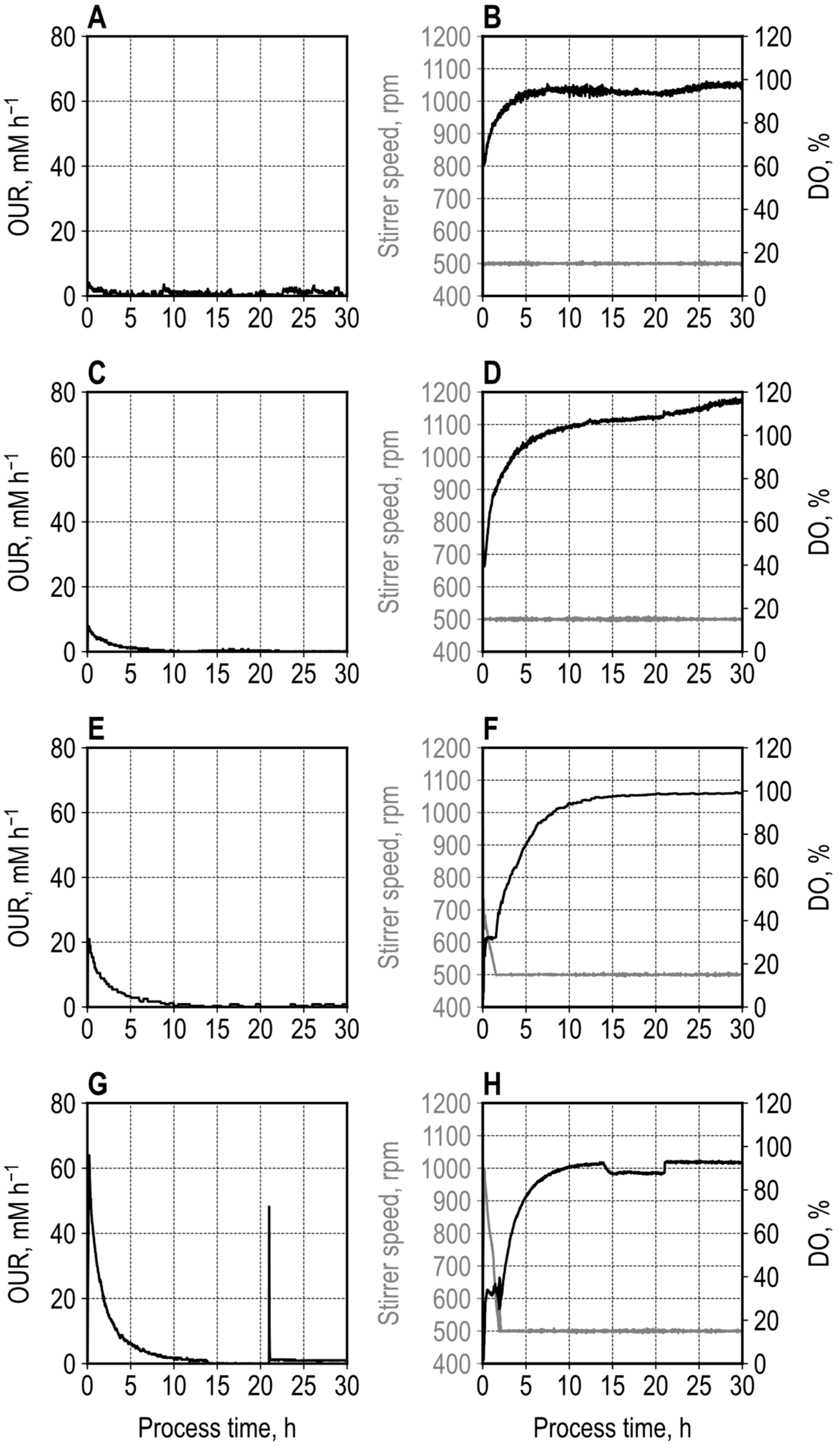

Figure S3. OUR, stirrer speed (grey), and DO concentration (black) in bio-oxidations with non-growing cells of *G. oxydans* BP9.1 pta-mGDH in STRs with different initial CDW concentrations: c_{X0} = 0.4 g L^{-1} **(A,B)**, c_{X0} = 0.7 g L^{-1} **(C,D)**, c_{X0} = 1.4 g L^{-1} **(E,F)**, and c_{X0} = 5.0 g L^{-1} **(G,H)** (c_{S0} = 45 g L^{-1}, pH 4.0, T = 30 °C, F_{air} = 2.0 vvm, V_R = 0.4 L, DO > 30% air saturation ensured by increasing stirrer speed, n = 500–1000 rpm).

https://doi.org/10.3390/biotech15020040

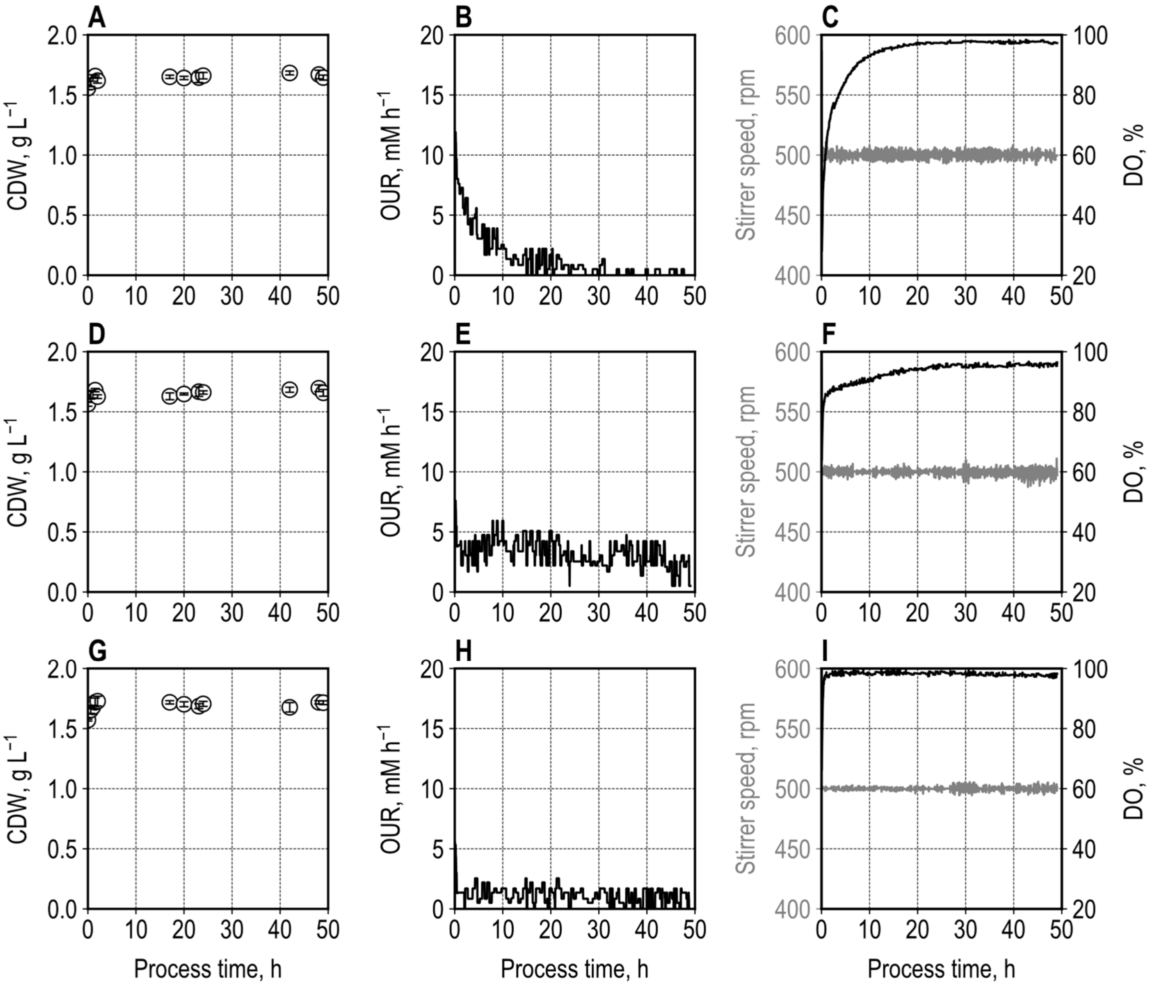

Figure S4. CDW concentration, OUR, stirrer speed (grey), and DO concentration (black) as a function of process time in bio-oxidations with non-growing cells of *G. oxydans* BP9.1 pta-mGDH in STRs at pH 4 **(A–C)**, pH 5 **(D–F)**, and pH 6 **(G–I).** Error bars in **(A,D,G)** represent the standard deviation of the OD measurements in technical triplicates (c_{S0} = 19 g L^{-1}, T = 30°C, F_{air} = 2.0 vvm, V_R = 0.5 L, DO > 30% air saturation).

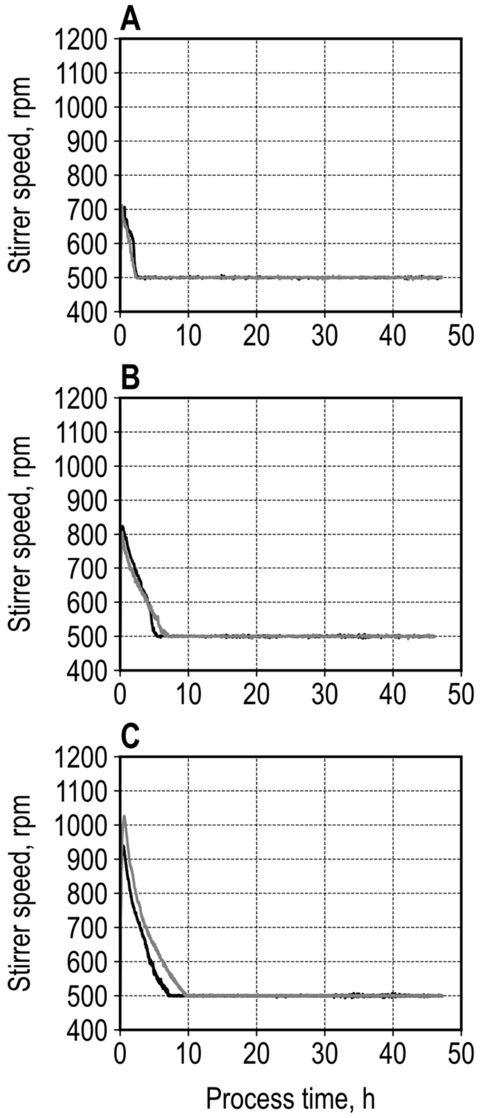

Figure S5. Stirrer speed as a function of process time in bio-oxidations with non-growing *G. oxydans* BP9.1 pta-mGDH in STRs. Different CDWs and initial GA concentrations were applied: CDW_0 = 2.86 ± 0.04 g L^{-1}, $c_{GA,0}$ = 48.6 ± 0.1 g L^{-1} **(A)**; CDW_0 = 2.72 ± 0.04 g L^{-1}, $c_{GA,0}$ = 93.9 ± 0.5 g L^{-1} **(B)**; CDW_0 = 3.44 ± 0.11 g L^{-1}, $c_{GA,0}$ = 93.8 ± 1.0 g L^{-1} **(C).** The batch processes were reproduced once, duplicates are shown in black and grey (pH = 5.0, T = 30 °C, F_{air} = 2.0 vvm, V_R = 0.4 L, DO > 30% air saturation ensured by increasing stirrer speed, *n* = 500–1030 rpm).
